# Nail growth arrest under low body temperature during hibernation

**DOI:** 10.1186/s12576-024-00919-2

**Published:** 2024-04-27

**Authors:** Taiga Ishimoto, Hideyuki Kosumi, Ken Natsuga, Yoshifumi Yamaguchi

**Affiliations:** 1 Laboratory of Biochemistry, Graduate School of Veterinary Medicine, Hokkaido University, Sapporo, Hokkaido Japan; 2 Department of Dermatology, Faculty of Medicine and Graduate School of Medicine, Hokkaido University, Sapporo, Hokkaido Japan; 3 Hibernation Metabolism, Physiology and Development Group, Institute of Low Temperature Science, Hokkaido University, Sapporo, Hokkaido Japan; 4 Graduate School of Environmental Science, Hokkaido University, Sapporo, Japan; 5 Inamori Research Institute for Science Fellowship (InaRIS), Kyoto, Japan; 6 Present Address: Department of Neuroscience for Metabolic Control, Graduate School of Medical Science, Kumamoto University, Kumamoto, Japan

**Keywords:** Hibernation, Nail growth, Stem cell, Nail matrix

## Abstract

Growth and differentiation are reduced or stopped during hibernation, an energy conserving strategy in harsh seasons by lowered metabolism and body temperature. However, few studies evaluated this in a same individual using a non-invasive method. In this study, we applied a non-invasive tracking method of the nail growth throughout the hibernation period in the same hibernating animals, the Syrian hamster (*Mesocricetus auratus*). We found that nail growth was markedly suppressed during the hibernation period but rapidly recovered by the exit from the hibernation period. Our data suggest that nail growth was arrested during deep torpor, a hypometabolic and hypothermic state, but recovered during periodic arousal, a euthermic phase. Consistent with this, nail stem cells located in the nail matrix did not exit the cell cycle in the deep torpor. Thus, hibernation stops nail growth in a body temperature-dependent manner.

## Background

The nails and claws in amniotes are homologous organs and play adaptive roles in many aspects of animal behaviors such as, holding or grasping foods, walking, scratching, digging, climbing, defensing and grooming. The nail contains a hard keratinized structure which is known as nail plate on the extensor side of the digits. The nail plate is located on each digit as skin appendages and protects the digit from physical damages. Nail growth is supported by nail stem cells that differentiate to form the nail plate in the nail matrix [1–3]. Aberrant nail plate production can cause pain in human, which can trigger gait problems [4]. Stem cells in nail matrix can be stressed with severe malnutrition [5], systemic disease [6, 7], or drugs [8], which impairs normal nail plate production.

One of the physiological phenomena in which animals are supposed to be severely stressed and nail growth is expected to be perturbed is hibernation (HIB). HIB is a survival strategy for surviving the cold season when food supply is limited or depleted. Among the various types of HIB observed in the animal kingdom, including ectotherms and endotherms, mammalian HIB is of interest because mammalian hibernators can actively reduce basal metabolism and thermogenesis, thereby being a severe hypothermic state in which the core body temperature (Tb) of small mammalian hibernators is often below 10 °C to conserve energy consumption in a cold season [9, 10]. This state is called deep torpor (DT) and lasts for several days or weeks. After several days of DT, Tb spontaneously returns to euthermia for a short period, often less than a day, which is called interbout arousal or periodic arousal (PA). Animals repeat the cycle of DT and PA many times during the HIB period and then quit the HIB and enter the post-HIB period. In many hibernators living in natural environments, this post-HIB period corresponds to spring when ambient temperature and photoperiod gradually become higher and longer, respectively. Interestingly, animals maintained in a constant laboratory condition spontaneously exit HIB after several months of HIB period and enter the post-HIB period without any environmental cues.

Previous studies on small mammalian hibernators suggest that during HIB period, cell proliferation is suppressed in various organs of hibernators [11, 12]. In arctic ground squirrels (*Citellus undulatus*), the mitotic index of intestinal epithelial cells decreases during DT and increases during PA [13]. In thirteen-lined ground squirrels (*Ictidomys tridecemlineatus*), the repair of muscle injury by satellite cells is delayed during HIB [14]. In Syrian hamsters (*Mesocricetus auratus*), adult neurogenesis decreases during DT [15]. Likewise, spermatogenesis is decreased and erythrocyte maturation is strongly inhibited during HIB [16]. However, to the best of our knowledge, there are no reports that track stem cell dynamics in the same individual during HIB.

In this study, to examine the hypothesis that nail growth is perturbed during HIB, we successively tracked the nail growth of a facultative hibernator, Syrian hamster, throughout the HIB period using a non-invasive method to measure nail growth, thereby revealing the dynamics of nail growth during HIB.

## Methods

### Animals and housing

8 weeks old Male Syrian hamsters were purchased from SLC, Inc., Japan, and housed as a single per cage with ad libitum access to diets (MR standard diet, Nihon Nosan, Japan) in a cold and short photoperiodic, winter-like condition (light: dark cycle = 8 h:16 h, lights on 10.00–18.00, ambient temperature = 5 °C) to induce hibernation, as previously described [17–19]. The onset of hibernation was detected comprehensively by the characteristic postures of the animals (rolled into ball), reduced activity, and consumption of food when the cages were changed. The sawdust method was used to detect animals that successfully hibernated; wood chips placed on the backs of hibernating individuals remained in place until the animals experienced periodic arousal. The hibernating animals used for nail marking were placed in cages whose gas concentration was monitored at 5 min intervals for several months by ARCO-2000 (ARCO system, Japan) [20] to judge whether nail marking affects the pattern of HIB as well as to measure duration of DT and PA. The age of the animals at the time of inspection for nail growth was as follows: cold-adapted group (CA) and interrupted hibernation group (interrupted HIB): 23–29 weeks old (*N* = 8 and 2, respectively), hibernation group (HIB): 26–31 weeks old (*N* = 3), and post-HIB group (post-HIB): 38–42 weeks old (*N* = 4). The animals were judged to be in the post-HIB period if more than one month had passed since the last HIB was observed and if their body weight had increased during this period.

### Nail marking

A 5% crystal violet solution was used to mark nail plate. The digits were defined as the first to fifth digits from the medial side, and the fourth digit on each side was used for nail marking according to a literature [21] with minor modification. Briefly, hamsters were anesthetized by inhalation of 3% isoflurane with oxygen to induce anesthesia, and 2% isoflurane with oxygen to maintain sedation. Under a stereomicroscope, the border of the nail plate and the nail epithelium was marked with a scalpel blade dipped in crystal violet solution, and markings were made weekly. In cases where hemorrhage was observed after marking, the mark was made one week later. Animals during the HIB period were marked when they were spontaneously aroused and were marked at intervals of 2 to 4 weeks. In the post-HIB group, the left and right third digit’s nail was used if the fourth digit’s nail had been injured by the previous marking.

### Assessment of nail growth

Marks on the nails were photographed and recorded at the same magnification using a stereomicroscope equipped with a digital CCD camera (M205FA, Leica). The distance from the proximal edge of the new mark to that of the old mark in the midline of the nail was measured from the obtained images at each time point using ImageJ (NIH, USA). Judgement of the edge of the mark was done by scar of the nail, wherein the crystal violet dye mark was most dense point, because the dye often dispersed around the scar, making it difficult to judge the real edge only by the dye color. Nail growth was defined as the increment of the distance. However, in the case of the HIB group, the distances between the two edges were too short to be accurately measured. We measured the distance from the nail epithelium to the proximal edge of the mark at each time point and used it to calculate the increment.

To determine duration of the euthermic PA period for normalizing nail growth during the HIB period by it, the oxygen consumption rate (OCR) obtained by ARCO-2000 was used. The changes of OCR precedes and well recapitulate that of Tb, as the OCR reflect basal metabolism and thermogenesis that causes Tb changes [22]. In this study, we defined each timepoint as follows: the starting timepoint of the euthermic PA period was the timepoint when the first peak of OCR after arousal from DT, and the end timepoint of the PA period was the last peak of OCR before the entrance into DT, during which the OCR continuously declined over 3 h. Total of the time spent in PA periods was used for dividing the total time during the HIB period (h) to derive the value of HIB/PA.

### Histology

The fifth digits of animals used for nail marking were cut with anesthesia, fixed in 10% formalin solution, decalcified with ethylenediaminetetraacetic acid, and processed for paraffin sections. Immunostaining was performed using antibodies against phosphor-Histone H3 (pH3) (06-570, Merck), PCNA (PC10, DAKO), anti-Ki67 antibody (ab16667, Abcam). For area measurement of the transitional zone and nail matrix zone, each zone was identified based on its morphology. For comparison between DT and PA, the right digit of a hibernating animal (DT) was cut to induce arousal, and 4 h later, at the timepoint when the animals was fully recovered to euthermia (PA), the left digit of the same animal was collected after anesthetization.

## Results

### Successive measurements of nail growth was achieved on the same individual hamster

**Fig. 1:**
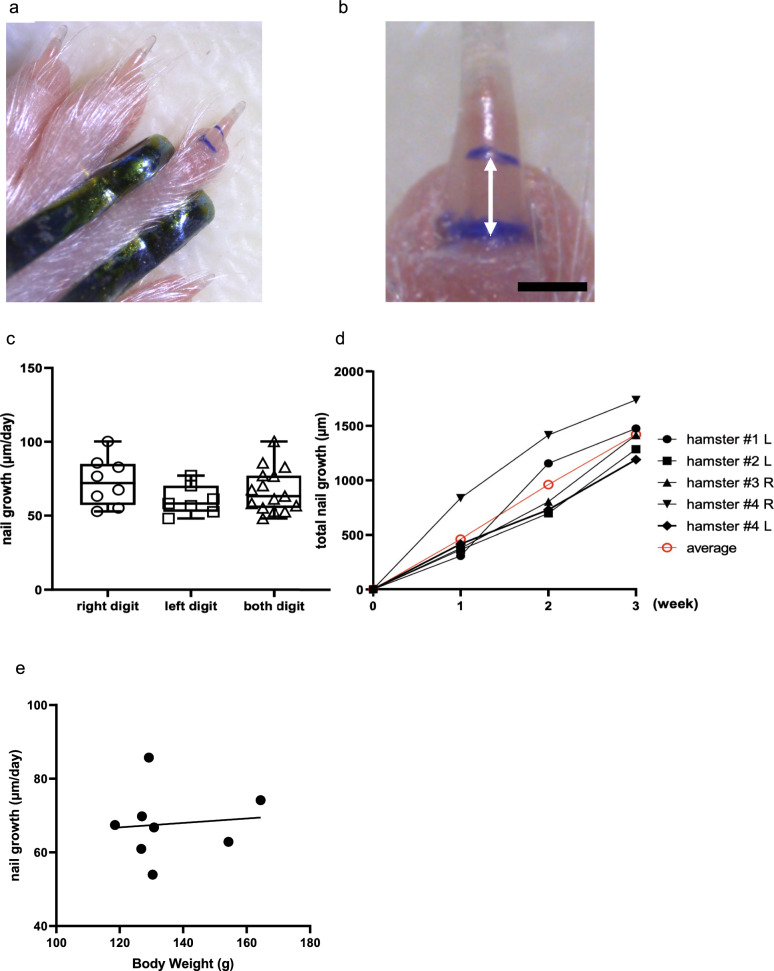
Evaluation of nail growth in Syrian hamsters. **a** Labeled nail in the fourth digit of the right forelimb of the Syrian hamster. **b** Measurement of nail growth by sequential labeling. Syrian hamster nails. The nails grew to 540 μm in 7 days in this case. Scale bar: 500 μm. **c** Nail growth of the fourth digit in one week in cold-acclimated (CA) hamsters. No differences in nail growth were observed between the right and left digits. Each point represents one digit, resulting two point from one individual in most cases. Nail growth in right digit: 73.0 ± 5.4 (*N* = 8), left digit: 60.6 ± 3.5 (*N* = 7), and sum of them: 67.2 ± 3.7 (*n* = 15 from total 8 animals). **d** Total nail growth during 1–3 weeks. The Y-axis indicates the sum of nail growth per week along the time-course labeling (X-axis) for each digit. Each point represents the nail length. **e** No correlation was found between body weight and nail growth per day. Each point represents one individual (*N* = 8). Nail growth per day was represented as a mean value of the right and left digit

First, we developed a method to evaluate nail growth during hibernation. We marked the distal end of the nail plate with crystal violet solution every week to measure nail growth (Fig. 1a, b) (see Materials and Methods for details). No significant difference was observed in nail growth between the right and left digits (Fig. 1c). We then used both the right and left digits for the measurement without distinction. Nail growth was almost constant per week (Fig. 1d), suggesting that damage caused by labeling, if any, did not affect nail growth. No correlation was observed between nail growth and body weight (Fig. 1e). These results suggest that the method used here is practical for successive measurements of nail growth in the same individual.

### The nail growth was suppressed during the HIB period

**Fig. 2:**
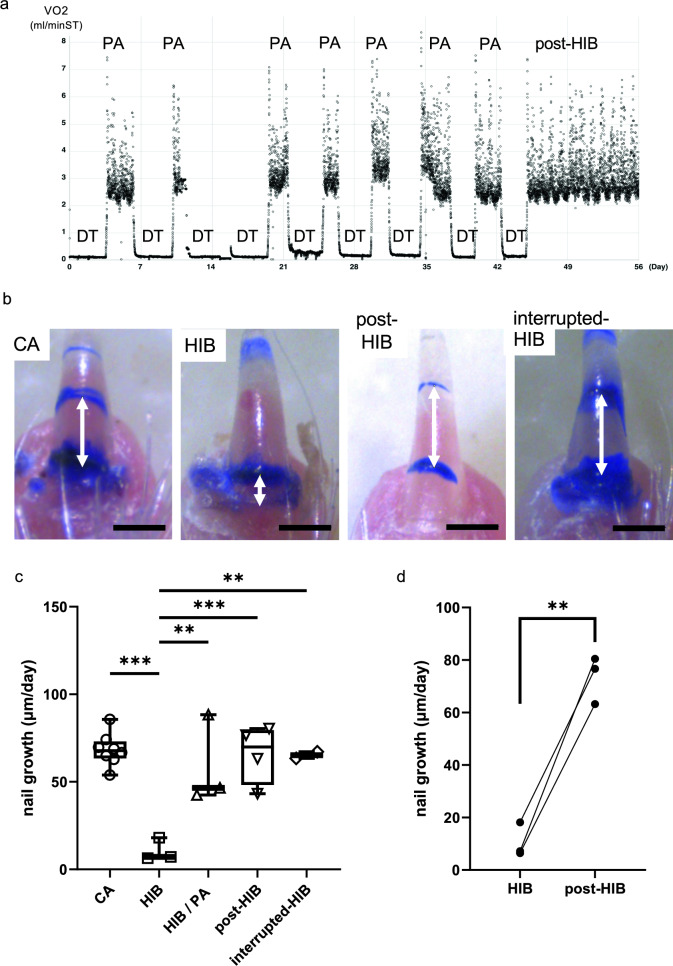
Comparison of daily nail growth during HIB. **a** Representative example of the oxygen consumption rate (OCR) of the hibernating Syrian hamster measured every 5 min (ml/minST). Each point represents the measured VO_2_ value. **b** Photographs of labeled nails. From left to right, the nail grew 627 μm after 14 days in the CA group, 253 μm after 26 days in the HIB group, 777 μm after 14 days in the post-HIB group, and 750 μm after 15 days in the interrupted HIB group. **c** Daily nail growth in the CA, HIB, HIB/PA, post-HIB, and interrupted HIB groups. Independent measurements of one finger of the left and right forelimbs per animal were done and each point represents the average of the two digits. CA (*n* = 8), HIB (*n* = 3), HIB/PA *(n* = 3), and post-HIB *(n* = 4). interrupted-HIB (*n* = 2). HIB/PA values were determined by dividing the growth during HIB by the period spent in PA. Only the HIB group exhibited a significant reduction in nail growth compared with the other groups (**p < 0.0005, ***p < 0.0001, Tukey–Kramer test). **d** Daily nail growth was recovered by the exit from the HIB to post-HIB in the same individuals. The average of nail growth in the left and right forelimbs was plotted for the hibernated animals. Each line represents a nail-growth transition (**p < 0.01, paired *t*-test)

Using the aforementioned method, we measured and compared the nail growth of hamsters at various stages of the hibernation cycle. Animals that had hibernated in the cold room were selected for nail growth measurement and set in chamber cages to monitor the oxygen consumption rate (OCR) in real time (Fig. 2a). Only hibernating animals exhibited significantly lower nail growth per day than animals in other states; cold-acclimated (CA) animals that were in the cold room but had never hibernated, Post-HIB animals that quit hibernation spontaneously in the cold room after several months of hibernation, and interrupted HIB animals that had hibernated before the nail labeling but stopped hibernation after it, possibly due to perturbation by the labeling procedure (Fig. 2b, c, Table 1). No significant differences in nail growth were observed among the CA, post-HIB, and interrupted HIB animals. Furthermore, comparison of nail growth between hibernating animals and Post-HIB animals in the same individuals indicated that the suppression of nail growth during the HIB period was canceled in the post-HIB period (Fig. 2d).

**Table 1:** Nail growth of each group

Group	CA	HIB	HIB/PA	Post-HIB	Interrupted-HIB
Average ± SE (μm/day)	68.4 ± 3.0	10.6 ± 3.1	59.1 ± 12.0	65.9 ± 7.3	65.3 ± 1.4
*n*	8	3	3	4	2

### Nail growth occurred at euthermic periodic arousal phase during hibernation

Next, we investigated the timing of nail growth suppression during HIB. When the nail growth during the HIB period was normalized by the time spent in the euthermic PA phase, the obtained values (HIB/PA) were not significantly different from the nail growth of the CA, post-HIB, and interrupted-HIB groups (Fig. 2c, Tables 1, 2). No significant difference was found between the post-HIB and normalized HIB/PA values (Table 1). These results suggest that nail growth occurs in the euthermic PA phase during HIB to an extent comparable to that in non-HIB and post-HIB phases, and that the suppression of nail growth is due to the hypothermic DT phase during HIB.

**Table 2:** Normalization of nail growth by PA duration

Sample no.	Hamster#5 R	Hamster#5 L	Hamster#6 R	Hamster#6 L	Hamster#7 R	Hamster#7 L
Measurement period (day)	30	15	36	36	26	26
Nail growth (μm)	420	333	307	153	120	253
Nail growth (μm/day)	14.0	22.2	8.5	4.3	4.6	9.7
PA period (h)	183	66	119	119	105	105
Correction of nail growth by PA period (μm/day)	55.1	121.6	62.0	31.0	27.3	57.7

### Proliferative potential is maintained in nail stem cells during HIB

**Fig. 3:**
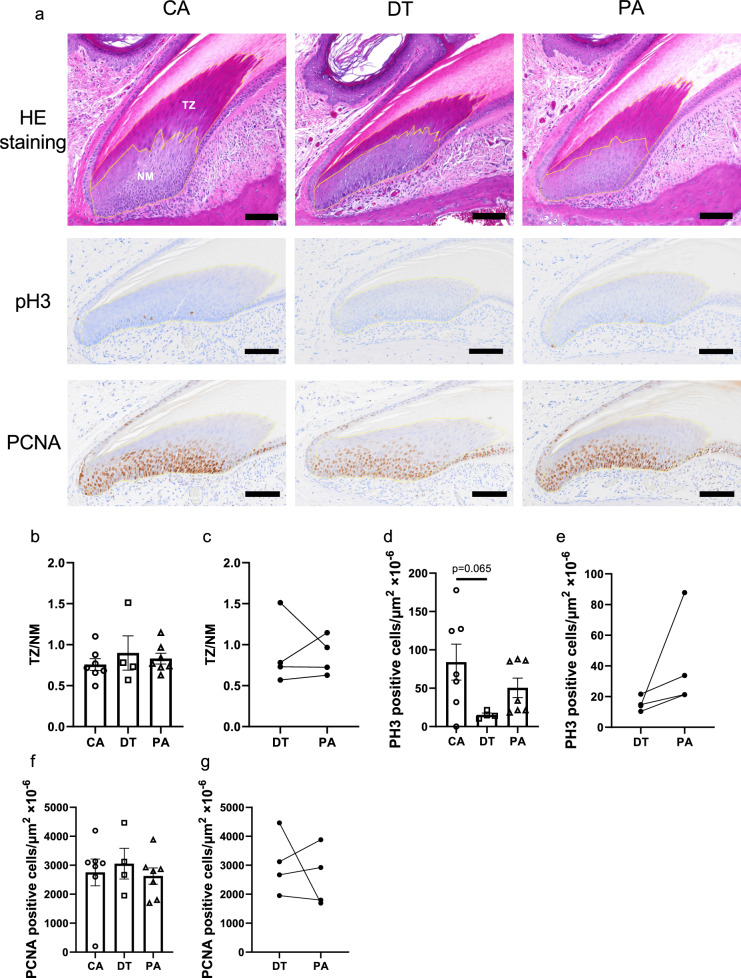
Evaluation for the proliferative potential of nail stem cell. **a** HE staining of the nail organ (top). *TZ* transitional zone. *NM* nail matrix zone. Immunostaining (brown) of pH3 (middle) and PCNA (bottom). Scale bars: 100 µm. **b** No significant difference in the ratio of TZ to NM among the groups (mean ± SE). Each point represents one individual. CA (*N* = 7), DT (*N* = 4), PA (*N* = 7). **c** The ratio of TZ to NM did not differ between DT and PA in the same individual (*N* = 4) (ns, paired *t*-test). **d**–**g** Quantification of the number of cells positive for pH3 or PCNA normalized to the NM area (mean ± SE). **d**–**f** Each point represents one individual. CA (*N* = 7), DT (*N* = 4), PA (*N* = 7) (Tukey–Kramer test). **e**–**g** The number of cells positive for pH3 or PCNA normalized to the NM area did not differ significantly between DT and PA in the same individual (*N* = 4) (ns, paired *t*-test)

Nail growth is achieved by nail plate formation through differentiation of nail matrix stem cells, and in the process, denucleation occurs at the transitional zone (TZ) located at the border of nail matrix and nail plate. To evaluate the denucleation of nail stem cells, we measured the area of the TZ and nail matrix zone (NM) (Fig. 3a). It is expected that the ratio of TZ to NM would increase if stem cell division is suppressed but differentiation (denucleation) is not. Conversely, the TZ/NM ratio would decrease if stem cell division is not suppressed but differentiation is. However, there were no significant differences in the TZ/NM ratio among all groups (DT, PA) (Fig. 3b,) or between DT and PA in the same individual (Fig. 3c). We also stained mitotic cells with phosphor-Histone H3 (pH3), a marker of cells at M phase. Cells positive for pH3 tended to decrease only in DT group (Fig. 3a, d). We then evaluated the proliferative potential of hamster nails. Nail organs were collected from each animal and stained with PCNA and Ki67, distinct markers for proliferative cells. There was no change in the density of PCNA or Ki67-positive cells in the nail matrix in all groups (DT and PA) compared with that in the CA group (Fig. 3a, f and data not shown). To gain insight into the changes in proliferative potential between DT and PA, nail organs were collected from the same individual and stained for the above markers. The number of pH3 tend to increase, though not significantly, in PA compared to DT (Fig. 3e), In contrast, no significant differences were found in the number of PCNA or Ki67-positive cells in the nail matrix between DT and PA in the same individual (Fig. 3g and data not shown). Collectively, these data suggest that nail stem cells in the NM maintain proliferative potential during HIB period and that mitosis may be arrested during DT.

## Discussion

In this study, we demonstrated that nail growth was suppressed in Syrian hamsters during the HIB period and recovered during the post-HIB period. Specifically, the length of the nail growth observed during the HIB period corresponded to that expected to occur only during the euthermic PA period. Furthermore, the nail growth observed in the interrupted HIB animals, whose HIB was terminated possibly due to the labeling procedure itself, was comparable to that in CA animals that did not hibernate under prolonged cold and short-photoperiodic, winter-like conditions. We also observed that there were no significant changes in the number of proliferative cells judging from PCNA staining. Interestingly, dividing cells positive for M-phase marker pH3 tended to decrease in the DT phase compared to the euthermic CA phases. These observation implicates that nail growth is arrested during the DT phase but is quickly restored at the euthermic PA phase due to the sustained potential of nail stem cell proliferation and differentiation. All the processes of nail growth, including nail stem cell division, differentiation and denucleation of the nail matrix, and growth into nail plates, are considered to be arrested under low body temperature, because many anabolic reactions mediated by enzymes are slowed down in such situation.

The data presented here are consistent with previous studies that the progression of cell cycle is arrested in DT in other organs of hibernators [12]. In the small intestine of the ground squirrel (*Citellus undulatus*), the mitotic index of intestinal epithelial cells decreases during DT and increases during PA [13]. In the brains of Syrian hamsters, uptake of BrdU is reduced during DT in the subventricular zone and hippocampal dentate gyrus, wherein adult neurogenesis takes place [15]. In the liver of the thirteen-lined ground squirrel (*Ictidomys tridecemlineatus),* it was reported that the expression of two cell cycle regulators, p15INK4b and p21CIP1, was upregulated upon DT [23]. p15INK4b inhibits the binding of cyclin D to Cdk 4/6 and p21CIP1 inhibits the binding of cyclin A to Cdk 2, thereby actively suppressing cell cycle progression. Compared to hibernators, cells of non-hibernators including humans and mice are vulnerable to cold [24, 25]. As such, it is difficult to examine long-term effect of cold on cell cycles in those species, but there are several reports demonstrating that cold exposure induces acute cell cycle arrest in non-hibernators. In human cancer cell lines, the transition from G2 to M phase of the cell cycle is temperature sensitive and vulnerable to low temperatures [26]. This observation is consistent with the idea that G2 arrest observed in hibernating intestinal epithelial cells might play a role in preventing cold-induced defects of cell division [13]. Thus, cell cycle arrest under low body temperature is likely a common feature to hibernators and non-hibernators. Future studies will be required to address whether the cell cycle arrest during DT is caused passively and solely by low temperature or accompanies unknown hibernator-specific regulated processes prior to or during the drop of body temperature.

Keeping the nail integrity and growth during HIB seems crucial for Syrian hamsters, since the animals are food-storing hibernators that stores and eat food at PA during HIB periods that can last for several months. Maintaining nail growth during HIB would be helpful for eating foods and digging the nest if necessary. In the case of human, abnormal nail growth and integrity is often observed in severely stressed condition. Because the nail is one of the few organs with potential regenerative capacity in mammals [27], understanding how nail growth arrest can be achieved without any abnormality in hibernators might lead to clinical applications of artificial hypometabolism and hypothermia in the treatment of nail and skin abnormalities or regenerative medicine.

## Data Availability

The datasets during and/or analyzed during the current study available from the corresponding author on reasonable request. Research data supporting this publication are available upon a reasonable request.
